# Positron Emission Tomography for Response Evaluation in Microenvironment-Targeted Anti-Cancer Therapy

**DOI:** 10.3390/biomedicines8090371

**Published:** 2020-09-22

**Authors:** Noboru Oriuchi, Shigeyasu Sugawara, Tohru Shiga

**Affiliations:** 1 Advanced Clinical Research Center, Fukushima Global Medical Science Center, Fukushima Medical University, Fukushima 960-1295, Japan; shige-s@fmu.ac.jp (S.S.); tshiga@fmu.ac.jp (T.S.); 2 Department of Nuclear Medicine, Fukushima Medical University Hospital, Fukushima 960-1295, Japan

**Keywords:** FDG-PET/CT, cancer stem cell, tumor microenvironment, immunotherapy, therapeutic evaluation, artificial intelligence, radiomics, theranostics

## Abstract

Therapeutic response is evaluated using the diameter of tumors and quantitative parameters of 2-[^18^F] fluoro-2-deoxy-d-glucose positron emission tomography (FDG-PET). Tumor response to molecular-targeted drugs and immune checkpoint inhibitors is different from conventional chemotherapy in terms of temporal metabolic alteration and morphological change after the therapy. Cancer stem cells, immunologically competent cells, and metabolism of cancer are considered targets of novel therapy. Accumulation of FDG reflects the glucose metabolism of cancer cells as well as immune cells in the tumor microenvironment, which differs among patients according to the individual immune function; however, FDG-PET could evaluate the viability of the tumor as a whole. On the other hand, specific imaging and cell tracking of cancer cell or immunological cell subsets does not elucidate tumor response in a complexed interaction in the tumor microenvironment. Considering tumor heterogeneity and individual variation in therapeutic response, a radiomics approach with quantitative features of multimodal images and deep learning algorithm with reference to pathologic and genetic data has the potential to improve response assessment for emerging cancer therapy.

## 1. Introduction

Positron emission tomography (PET) has become an indispensable procedure for the initial assessment and post-therapeutic evaluation in clinical oncology, using dedicated radiopharmaceuticals targeting cellular metabolism and tumor-specific receptors [1]. PET as a means of molecular-based imaging is able to characterize biological processes associated with disease progression and therapeutic response quantitatively at the cellular and molecular levels. The outcome of a therapy cannot be interpreted properly without a surrogate biomarker to assess the efficacy of every therapeutic modality.

Therapeutic response is objectively evaluable by means of imaging. Conventional response evaluation criteria use morphological parameters; on the other hand, 2-[^18^F] fluoro-2-deoxy-d-glucose (FDG)-PET-based criteria use metabolic parameters. Histological response to anti-cancer therapy depends on the therapeutic modalities; cancer immunotherapy shows the distinctive phenomenon of immune-related tumor responses. Emerging observational data of immune-related response patterns have determined modification of the conventional response criteria. The current approaches to anti-cancer therapy target the tumor microenvironment as well as anti-tumor immunity.

In this review, we summarize the current understanding of the tumor microenvironment, focusing on metabolism, cancer stem cells, chemokine receptors, and immune mechanisms, all of which are targets of therapy. Molecular imaging may have promise to address therapeutic response and toxicity evaluation to provide useful information for the benefit of novel anti-cancer therapy. PET in the assessment of individual therapeutic effectiveness plays a definitive role in personalized therapeutic strategies within the framework of precision medicine.

## 2. Glucose Metabolism of Cancer and FDG-PET

It has been appreciated for nearly 100 years that cancer cells are metabolically distinct from other cells. All cells fundamentally require nutrients to meet metabolic demands for energy generation and biosynthesis. Metabolic demands of cell proliferation, differentiation, and biosynthesis of proteins, lipids, and nucleotides are different in tumor cells.

Elevated glucose uptake and cellular metabolism were thought to be the biochemical characteristics of cancer [2]. FDG-PET could disclose a high glycolytic rate and pyruvate oxidation in the mitochondria, depending on the cell proliferation. These altered metabolisms, including metabolic switch from aerobic to anaerobic glycolysis, are known as the Warburg effect [3,4]. The function of the Warburg effect has been simply understood as a metabolic switch, but a breakthrough to explain the Warburg effect regarding cancer metabolism in vivo has taken place recently [5,6,7,8].

Tumor hypoxia is known to be the most important factor to account for biological aggressiveness and resistance to chemotherapy and radiotherapy through the expression of multidrug resistance 1 (MDR1) and cell cycle arrest [8,9]. Accelerated proliferation and metabolism of cancer cells lead to an imbalance in the form of insufficient oxygen supply in relation to oxygen demand in solid tumors [10,11]. Anti-neoplastic drugs and ionizing radiation have effects on oxygen to generate reactive oxygen species (ROS) in cancer cells, causing oxidative stress, which results in apoptosis. However, cancer cells can survive in the hypoxic area, which is usually seen at 100 μm from tumor vessels, because of the decreased generation of ROS [12]. In the area of hypoxia, a transcription factor, hypoxia-inducible factor 1 (HIF-1), is activated to induce the expression of various genes responsible for adaption to hypoxic metabolism from oxidative phosphorylation to glycolytic ATP production, explained by the Warburg effect, as mentioned above [13,14], invasion and metastases of cancer cells through the formation of pre-metabolic niche and epithelial–mesenchymal transition (EMT) to escape from hypoxia [15,16], increased erythropoiesis through upregulation of erythropoietin, and angiogenesis to reoxygenation of hypoxic area [17]. An α-subunit of HIF-1 (HIF-1α) induces expression of glucose transporter 1 and glycolytic enzymes to increase glucose uptake and anaerobic glycolysis to compensate for ATP production [18,19]. FDG-PET can therefore evaluate tumor aggressiveness and resistance to chemotherapy and radiotherapy by detecting increased glucose metabolism and is a possible therapeutic marker to monitor responses.

## 3. Quantitative Parameters for Response Evaluation with PET

The ability to obtain quantitative information with tracer uptake in the tumor would be relevant for the evaluation of therapy monitoring with PET. Utility of quantitative value depends on the tracer used for the PET study. Uptake and retention of the tracers are greatly varied depending on the interaction between biochemical properties of tracers and biological characteristics of cancer cells, such as avidity, specificity, and intracellular metabolism as well as the specific activity of the tracer. The widely used PET tracer is FDG. Uptake of FDG depends on the glucose metabolism characterized by the increased expression of glucose transporters and glycolytic enzymes whose proportions are excessive to oxidize pyruvate in the tumor.

Initial uptake and time-series changes after therapeutic intervention vary greatly. Standardized uptake value (SUV) is a normalized measure of radiotracer uptake, defined as the ratio of the radioactivity concentration in a defined region (MBq/mL) to the injected radioactivity that is corrected for total body mass (MBq/g). The maximal values of SUV, expressed as SUVmax and SUVpeak, are used for evaluating tumor aggressiveness and as prognostic markers of the tumor [20,21,22,23,24,25,26]. SUV is a standard measure in FDG-PET which is representative of tumor viability and aggressiveness. Although controversy about SUVmax has been shown regarding its accuracy and reproducibility, use of SUVmax or other measurements may depend on the purpose of the study and other clinical situations. SUVmax is thought to be a mean value of approximately 3 mm cubic voxel in the region of interest (ROI); it contains approximately 10^7^ cells [27]. SUVmax is then an averaged value of the tracer uptake in sufficient numbers of cancer cells and other cells in the most metabolically aggressive part of the potentially heterogeneous tumor.

SUVpeak is determined by averaging the image data within a 12 mm-diameter spheric ROI that is positioned within the tumor so as to maximize the enclosed average. Therefore, SUVmax is less susceptible to partial volume effects than other indices. SUV is typically normalized to total body mass; however, because there is little accumulation of FDG in white adipose tissue (WAT) in the fasting state, obese participants tend to have higher SUV in the organs except WAT. On the contrary, SUV of the target tissue in lean patients will be lower than that in heavier patients. Therefore, normalized SUV using the lean body mass (SUVlean) instead of total body mass (SUV) has been proposed [28].

Total lesion glycolysis (TLG) and metabolic tumor volume (MTV) are more complicated values that reflect both tracer uptake and the volume of the tumor. They have been recognized as useful indictors for various tumors; however, these values depend on the tumor volume as well as the selected threshold value of SUV and are mainly used for evaluating therapeutic response [29,30,31,32,33]. A recent meta-analysis showed that TLG and MTV were better predictors of treatment outcomes than SUVmax and SUVmean in lung cancer [34].

## 4. Therapeutic Monitoring with FDG-PET

Therapeutic response was originally evaluated by the post-therapeutic changes in the unidimensional longest diameter measured by conventional imaging, such as computed tomography (CT), that was fundamentally followed by the revised criteria in 2009 (Table 1) [35]. PET/CT-based response evaluation criteria have been shown to be relevant in cases with patients undergoing chemotherapy and especially in cases with molecular-targeted therapy. There are currently two sets of criteria available for response evaluation: the criteria developed by the European Organization for Research and Treatment of Cancer (EORTC) and PET Response Criteria in Solid Tumors (PERCIST) (Table 1) [36,37].

Therapeutic response evaluated with FDG-PET varied according to the therapeutic modalities and drugs. In the case of antineoplastic drug therapy, conventional chemotherapeutic agents, molecular targeted drugs, and immune checkpoint inhibitors have specific changes in FDG uptake which depend on the metabolic alteration of glucose after treatments. Metabolic changes occur in advance of volume reduction in molecular targeted therapy. An initial preliminary study suggested the usefulness of FDG-PET for the early prediction of gefitinib efficacy for the treatment of non-small cell lung cancer (NSCLC). The response to gefitinib may be predicted by the 60% metabolic decrease as early as two days after initiation of the treatment [38]. This knowledge reminded us of the need for relevant response evaluation methods [39,40].

Immune checkpoint inhibitors have experienced a breakthrough in the treatment outcome in cancer patients through the ability of the host immune system to eradicate malignant cells with limited systemic toxicity. Treatments with immune checkpoint inhibitors include antibodies against the cytotoxic T-lymphocyte associated protein 4 (CTLA-4), the programmed cell death receptor 1 (PD-1), or its ligand (PD-L1). For example, anti-PD-1 antibody is effective for many types of cancer including NSCLC [41]. Although their efficacy is outstanding even for patients with chemotherapy resistance, an established biomarker for predicting therapeutic efficacy is still underway, because the interaction between tumor cells and host immune cells that play various roles in anti-tumor immunity is complicated. This suggests that FDG-PET may reflect therapeutic efficacy by the immune checkpoint inhibitors, considering the results of a recent study that indicated that FDG uptake after administration of nivolumab was an independent prognostic factor, and PD-L1 expression or plasma concentration of nivolumab was not a predictor of early therapeutic efficacy [42]. FDG-PET is supposed to be useful for monitoring early response to anti-PD-1 antibody, and several reports in the literature show the value of monitoring the anti-tumor efficacy of immune-checkpoint inhibitors; however, optimal timing to evaluate efficacy remains to be addressed [43,44]. Increased clinical use of immune checkpoint inhibitors determined the necessity of creating appropriate evaluation criteria using FDG-PET.

## 5. Machine Learning for Imaging Cancer Heterogeneity and Interpretation

**Figure 1:**
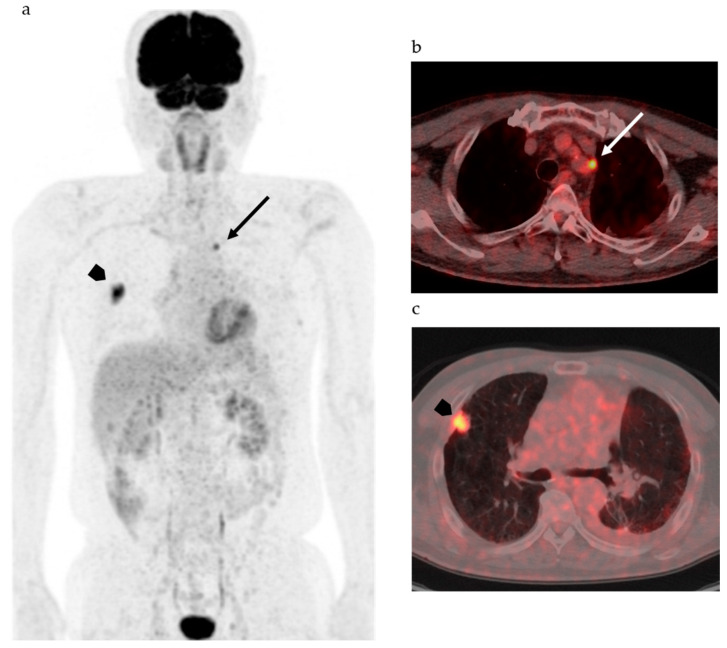
2-[^18^F] fluoro-2-deoxy-d-glucose positron emission tomography/computed tomography (FDG-PET/CT) of patients with non-small cell lung cancer. (**a**) Maximum intense projection image shows abnormal FDG uptake in the left upper mediastinum (arrow) and the right lung (arrowhead). (**b**) PET/CT shows increased FDG uptake in the lymph node adjacent to the left subclavian artery at the level of the upper mediastinum (arrow). (**c**) High FDG uptake is seen in the nodule at the right upper lobe (arrow head).

Figure 1 shows FDG-PET/CT images of a patient with NSCLC. Two foci of increased nodular FDG uptake are seen in the upper lobe of the right lung and the upper mediastinum on the left side. These lesions show SUVmax of 7.2 and 4.8, respectively. Do these images provide the radiologist with sufficient information for correct interpretation? Radiologists cannot diagnose correctly without additional information about clinical history, because every radiologist knows that the most common sites of metastasis of lung cancer are ipsilateral hilar and mediastinal lymph nodes and that metastasis to the contralateral mediastinal lymph node usually occurs after ipsilateral mediastinal lymph nodes metastases [45]. Information about past history of left lung cancer with T3N1M0, stage IIIA, for which left upper lobe segmentectomy and lymph node dissection was performed 2 years before, is a clue for correct diagnosis. The patient had undergone surgery followed by chemotherapy on the basis of correct diagnosis. How accurately can FDG-PET images estimate the efficacy of chemotherapy and prognosis of this patient? Do quantitative parameters help to predict response to chemotherapy and prognosis?

Considering the heterogeneity of tumor tissue, more sophisticated indexes surpassing SUV and other parameters as well as diagnostic algorithms are needed to accurately classify the tumor on the basis of biological malignancy, effectiveness of various types of therapy, and prognosis of patients. Recent advancements in computer science and artificial intelligence (AI) have shown the possibility for machine learning systems to take on the practice of radiology, which was previously thought to be limited to human radiologists. AI including machine learning technologies has the potential to transform radiological imaging by using the vast amount of clinical data including pathologic and genetic examinations to automate the integrated diagnostic radiology workflow and diagnosis. Machine learning algorithms, such as random forests, support vector machines, and artificial neural networks, have been used for classification of images by training with input data set and knowledge, and then the best model is applied for the prediction of pathophysiology. Due to deep learning and convolutional neural networks (CNN), the capability to learn and master given tasks to perform computer-aided diagnosis (CAD) has made remarkable advances in clinical radiology in the past decade [46,47]. Wang et al. have suggested that the performance of CNN from FDG PET/CT images is comparable to the best classical machine learning and human radiologists and that CNN is more convenient and objective than the classical methods, because it does not need tumor segmentation, feature selection, or texture features for classifying mediastinal lymph node metastasis in patients with NSCLC [46]. They also suggested that the performance of the CNN would be improved by incorporating diagnostic features like SUV and tumor size [46]. For example, in mediastinal lymph node metastasis in patients with NSCLC, accurate diagnosis is a challenge, as indicated in Figure 1; however, lymph node metastasis evaluated by FDG uptake has been reported to be prognostic as compared with pathological lymph node metastasis [48]. Therefore, diagnosis of lymph node status during diagnostic work up is of the utmost importance.

Machine learning is already being applied in the practice of radiology, including in the field of mammography. There have been many papers describing a performance level in lesion detection similar to that of experienced radiologists [49,50,51]. CAD was approved by the Food and Drug Administration (FDA) and has been used for mammography in radiology practices [52]; however, improvement of the diagnostic ability has not been satisfactory, and the majority of radiologists have rarely changed their reports as a result of findings generated by CAD [53,54,55]. Machine learning has been reported to be unlikely to replace radiologists but will provide quantitative tools to increase the value of imaging as a biomarker including therapeutic response evaluation [56]. Recently, radiology professionals have reminded that AI algorithms must be as safe and effective as the physician by rigorous testing, longitudinal surveillance, and investigation of oversight mechanisms to ensure generalizability across patients as well as variable imaging and imaging protocols [57]. However, radiologists cannot disregard autonomous radiology AI, because AI can tirelessly improve the image reading capacity and may drastically acquire interpretation capabilities if AI can incorporate available medical information and contextual integration of data that would typically be identified during physician interpretation in order to render a medical judgement.

On the basis of considering tumor heterogeneity, texture analysis has been explored, especially in the field of nuclear medicine [58,59,60,61]. The most exciting part of machine learning in medical imaging would be to extract patterns that are beyond human perception and classification due to the application of deep learning for diagnostic algorithms [62,63]. Radiologists should seek to work alongside AI in the future.

## 6. Radiomics for Diagnosis and Assessment of Current Therapy

Radiomics is a quantitative image analysis method that aims to correlate large-scale imaging data to clinical and biological endpoints [64]. The radiomics approach combines quantitative imaging features, called “radiomic features”, about size, shape, intensity, and texture with a machine learning algorithm to automatically contour the tumor lesion and diagnose qualitatively [58,65,66]. Machine and deep learning-based models have the potential to improve diagnosis, staging, and response assessment towards translation for more personalized treatment in oncology. For example, a study on radiomic features from FDG-PET and contrast enhanced CT features correlated with ^18^F-FMISO tumor-to-blood ratio detected the presence of hypoxia in head and neck cancer and provided a potential biomarker useful to microenvironment-oriented personalized treatment [67]. Associations between radiomic phenotype and gene expression were found in a variety of cancers [68,69,70,71]. In patients with lung adenocarcinoma, prediction of epidermal growth factor receptor (EGFR) mutation status by CT-based radiomic features has been reported, and another study revealed that the combination of CT imaging parameters and clinical features could provide diagnostic benefit to identify anaplastic lymphoma kinase (ALK), c-ros oncogene 1 (ROS1), and rearranged during transfection (RET) fusions [72,73]. Genotypic information is important in clinical practice for therapeutic decision making, such as using EGFR-tyrosine kinase inhibitors (TKIs) and crizotinib [74,75,76]. Because biopsy-based assays provide limited characterization of the tumor, the harvested samples may not always represent the heterogeneity of the tumor [77,78]. Many studies have shown the relevant role of a radiomics-based biomarker named the “radiomic signature” from various imaging modalities for the prediction of therapeutic response and prognosis after various therapeutic options, such as radiotherapy, chemotherapy, molecular-targeted therapy, and immunotherapy [76,79,80,81,82,83,84]. Patient selection on the basis of predicting responsiveness and toxicity is critical for novel therapeutic modalities, because these therapies are remarkably effective for a limited cohort, with the main drawbacks being its cost and the possibility of severe toxicities. Therefore, precise predictors of response to treatment are urgently needed for every treatment. In a retrospective multicohort study in patients with advanced solid tumors, a radiomic signature of CD8 cells, which is a characteristic phenotype of immune cells infiltrating the immune-inflamed tumors, was validated to infer clinical outcomes of patients treated with immunotherapy with anti-PD-1 or anti-programmed cell death ligand 1 (PD-L1) therapy [85]. Radiomics signatures can help to identify the development of side effects. A study on radiomics-based assessment showed dose and fraction-dependent radiation in lung injury after stereotactic body radiotherapy for lung cancer [86]. Radiomic assessment of predicting side effects and detecting recurrence has been performed mainly with CT and magnetic resonance imaging (MRI). Radiomics with FDG-PET and PET with new tracers will be examined for post-therapy assessment due to the merit of imaging molecular and metabolic mechanisms [87,88].

Although research in the field of radiomics has drastically progressed over the past decade, the refinement of clinical workflow and decision making through prospective clinical trials and standardization of diagnostic procedures are required to be used for personalizing the management of cancer.

## 7. Cancer Metabolomics as a Target of Therapy and Response Evaluation with PET

The disruption of tissue homeostasis induces critical changes in the cellular metabolism and function of both stromal and immune cells in the so-called tumor ecosystem. Therefore, cancer metabolism-targeted chemotherapeutic agents against nucleotide synthesis, such as 5-fluorouracil and gemcitabine, have been used in the traditional mainstream of cancer therapy. On the other hand, amino acid metabolism-targeted therapy with l-asparaginase has been performed for acute lymphoblastic leukemia (ALL), and several clinical trials have been done [89,90]. Cancer cells adapt metabolism to support tumor growth. Specificity of metabolic preferences was thought to provide possibilities of imaging tumor, prediction of prognosis, and selection of treatment option; however, recent studies disclosed that the metabolic phenotype is not unique to cancer cells but instead reflects the characteristics of proliferating cells, such as those in the immune system [91,92]. The clinical utility of FDG-PET is a result of its ability to reflect increased glucose consumption in a broad spectrum of proliferating and biosynthetic cells. Therefore, FDG-PET can detect not only malignant tumors but also some types of benign tumors, granulomas, inflammatory focuses, and autoimmune diseases.

In order to image specifically the malignant tumors, PET for targeting neoplastic cells has been evaluated by many kinds of radiopharmaceuticals, such as ^18^F-labeled amino acids [1,93,94,95]. One of the tumor-specific tracers for PET is 3-[^18^F] fluoro-l-α-methyltyrosine (^18^F-FAMT) [96]. ^18^F-FAMT is transported by l-type amino acid transporter (LAT-1), which is specifically expressed on a variety of cancer cells [97]. There have been many reports showing the utility of ^18^F-FAMT PET for evaluating prognosis and therapeutic response [98,99,100]. Therefore, inhibition of LAT1 has been considered for a wide range of anti-cancer therapies [101,102]. Glutamine is another fuel besides glucose that contributes to core metabolic functions and is primary nitrogen source for DNA replication in cancer cells [103]. Asparagine is biosynthesized with glutamate and nitrogen, and then asparagine itself promotes cell proliferation as well as cell survival in the absence of glutamine [104]. Therefore, l-asparaginase is used to reduce the blood asparagine levels and induce apoptosis of ALL cells as mentioned above [89,90,105]. However, the ability of cancer cells to use a panoply of energy sources by autophagic degradation of macromolecules is a negative factor for starving strategy in cancer therapy [106]. In addition, cellular metabolism depends on cell lineage, and the environment can significantly affect cellular metabolism [107,108]. Recent studies have shown the role of mitochondrial function in enabling therapeutic resistance and suggested mitochondrial inhibition as a promising therapy to overcome MDR [109,110,111].

Recent studies disclosed that autophagy is closely related with non-apoptotic cell death, called autophagic cell death, besides the conventional understanding of its fundamental role as a resistant mechanism against stress environments including hypoxia, chemotherapy, radiotherapy, as well as starvation. Regulation of autophagy is considered as a new treatment of malignant tumors.

## 8. Response Evaluation of Immune Checkpoint Inhibitor Therapy with PET

Although FDG-PET can provide practical information on responses in patients treated with anti-neoplastic drugs, immunotherapy, and radiotherapy, several limitations for FDG-PET have been raised to be addressed. FDG is highly accumulated in inflammatory tissues owing to the increased glycolysis in the infiltrated inflammatory cells. Activated T cells and recruited macrophages and neutrophils as well as lymphocytes show high uptake of FDG after immunotherapy, resulting in false-positive judgement in evaluating effective therapeutic response. The distinctive biologic mechanisms of immune checkpoint blockade cause unique response phenomena in the tumor after an initial enlargement called pseudoprogression [112]. Conventional Response Evaluation Criteria in Solid Tumors (RECIST) defines this as progression at the time of the initial enlargement that is thought to be owing to the infiltration of T cells and other inflammatory cells into the tumor, rather than proliferation of tumor cells [112,113,114]. These observations facilitated the modification of RECIST to accurately define the current immune-related tumor responses, irRECIST and iRECIST (Table 1) [115,116]. These criteria have been examined extensively, but the clinical significance of these criteria remains to be validated by prospective clinical trials, considering the presence of and temporal changes in the pseudoprogression.

A recent study revealed that the uptake of ^18^F-FAMT was significantly correlated with PD-L1 expression in patients with advanced NSCLC [100]. Moreover, emerging data have suggested certain utility for immune checkpoint molecule-specific PET that visualizes the key molecules of immune-checkpoint pathways and cellular immune responses using radiolabeled antibodies against PD-1, PD-L1, and CTLA-4 [117,118,119]. Novel PET imaging with engineered antibody-based PD-L1 antagonist conjugated with ^64^Cu-DOTA- and ^68^Ga-NOTA-labeled CD8^+^ T cell-targeted peptide have demonstrated early uptake in the tumor with favorable tumor-to-background ratios and the uptake reflecting tumor response to PD-1 inhibitor plus CTLA-4 inhibitor therapy in mouse xenograft models, respectively, and have seemed to overcome limitation of radioimmunodetection with conventional antibodies [120,121,122].

Cancer immunotherapy using immune checkpoint inhibitors has emerged as an effective therapeutic option for a variety of advanced cancers in the past decade. Because cellular metabolism is a critical determinant of the viability of cancer cells and responding immune cells, and the tumor microenvironments characterized by acidic, hypoxic, or depleted critical nutrients, the microenvironment is regarded as a novel target of cancer therapy [123,124]. Moreover, immunological reaction differs among patients according to the individual immune function. Since FDG uptake reflects glucose metabolism of cancer cells and immunologically competent cells in the tumor microenvironment as well, FDG-PET response evaluation criteria, such as EORTC and PERCIST, must be revised to conform to the temporal alteration of glucose metabolism after administration of various anti-cancer drugs.

## 9. Tumor Microenvironment and Cancer Stem Cells as a Target of Therapy

Failure of conventional anti-cancer therapy is closely related to the heterogeneity of tumors. A biochemical feature of cancer heterogeneity is considered the microenvironment that consists of cancer cells, immune cells, fibroblasts, and cancer stem cells (CSCs) [125]. CSCs have been reported as the cells of origin in cancer and are responsible for maintaining tumor heterogeneity and plasticity, fueling tumor proliferation and therapy resistance [126,127]. The concept of neoplastic stem cells may provide explanations for the failure of various anti-cancer therapies [127]. Secondary resistance occurs against every anti-cancer drug therapy and radiotherapy for cancer to survive, because CSCs have inhibitory effects on oxidative stress induced by ROS produced by chemotherapy and radiotherapy. HIF-1 has an important role in maintaining the stem cell phenotype and regulating the differentiation of CSCs in the hypoxic niche of the tumor microenvironment. CSCs are suggested as a potential source of resistance to different types of anti-neoplastic drugs. On the basis of HIF-1 dependent biological mechanisms such as reoxygenation in the peri-necrotic area, the possibility of HIF-1-targeted therapy has been shown to overcome the resistance to radiotherapy [128].

Metastasis of epithelial tumors is a complex process involving the enhancement of invasive properties to facilitate extravasation, liberation of circulating tumor cells (CTCs) in the blood, colonization to the secondary organs, and growth to become overt metastasis [129]. EMT is considered a principal mechanism of the metastatic cascade, providing tumor cells with the ability to adapt to different microenvironments in the colonized organs [130]. Therefore, detecting and characterizing CTCs helps in guiding early treatment decisions before the detection of overt metastases and assessing therapeutic efficacy [129,131]. CTCs are also considered as potential therapeutic targets [132].

Traditional anti-cancer drugs act on proliferating neoplastic cells by interfering with cell division and as molecular drivers for cell growth and tumorigenesis, resulting in the development of resistance in many tumors. Intrinsic resistance to both cytoreductive and cytostatic drugs has also been reported [133]. Moreover, CSCs have been reported to develop acquired drug resistance and differentiate into heterogeneous cellularity by producing more malignant subclones [134]. Clinical trials have indicated that circulating CSCs detected after the effective chemotherapy are associated with an increased risk of metastases that result in poor prognosis [135]. These findings are in line with the need for new treatment strategies focusing on the elimination of CSCs through which therapeutic efficiency and prognosis could be improved [136]. Therefore, considerable effort has been devoted to identifying biomarkers for CSC-targeted anti-cancer therapy; however, there is no effective therapy to eradicate CSCs or at least to inhibit their proliferation. Many attempts have been made to cope with CSCs in the context of the tumor microenvironment. For example, recent studies have shown that autophagy inhibitor leads to reduce tumor growth and invasion [137,138]. Inhibition of fatty acid and glutamine metabolism in vitro has been shown to reduce spheroid formation and stemness gene signature with radiation sensitization, respectively [104,139].

CSCs are thought to be responsible for proliferation and therapeutic limitation in many malignant tumors. Therefore, noninvasive imaging to visualize CSCs is required for diagnosis and monitoring after therapy. Initial studies with hypoxia imaging revealed that hypoxia-seeking agents such as ^64^Cu-diacetyl-bis (N4-methylthiosemicarbazone) (^64^Cu-ATSM) could evaluate CD133^+^ cells in hypovascular regions of murine lung and colon cancer models [140,141]. A recent study using S-2-(4-isothiocyanatobenzyl)-1,4,7-triazacyclononane-1,4,7-triacetic acid (NOTA) as the ^64^Cu chelator labeled with AC133 monoclonal antibody (^64^Cu-NOTA-AC133 mAb) for PET and AC133 mAb-based near-infrared fluorescence molecular tomography has indicated CSC monitoring through imaging CD133 expression of stem and progenitor cells in many types of malignant tumors of human origin [142,143]. In this study, the authors show that differences in PET radioactivity correlate with histopathological differences in the cellularity. Noninvasive PET imaging seems preferable to monitor CSCs in tumors after CSC-targeted anti-cancer therapy. However, there is no clinical PET procedure capable of high-sensitivity monitoring of CSCs.

## 10. Biochemical Properties of C-X-C Chemokine Receptor Type 4 and Cancer Immunotherapy

Chemokines and their receptors were originally identified as mediators of the inflammatory process; however, accumulated evidence clarified that they have a crucial role in tumor initiation and progression as the key factors linking cancer cells and stromal cells in the microenvironment for tumor growth and metastasis [144]. A previous study showed that expression of cytokine C-X-C chemokine ligand 12 (CXCL12), known as stromal-derived factor 1 (SDF-1), was the most significant biomarker predictive of survival on the basis of multiple microarray analyses measuring the expression of ~17,000 genes and 341 miRNAs across 2129 ovarian cancer samples [145]. CXCL12 can activate various signal pathways of cancer cells to promote proliferation, infiltration and angiogenesis and suppress apoptosis [144]. SDF-1/C-X-C chemokine receptor type 4 (CXCR4) is known to increase EMT to facilitate invasion and metastasis. Thus, CXCL12 and its receptor CXCR4 were thought to represent a promising therapy targeting the link between cancer cells and stromal cells [144].

Recently, the function of CXCR4 in the cancer immune cycle has been extensively addressed. CXCR4 plays a major role in immunological processes. The CXCR12/CXCR4 axis has influence on intratumor immune cell subsets and anti-tumor immune response. In the cancer microenvironment, EMT induces differentiation of tumor-associated macrophages (TAM) and regulatory T cells (Treg) through mesenchymal stem cells and cancer-associated fibroblasts. TAM and Treg are then induced to secrete transforming growth factor-β (TGF-β) and interleukin 10 and facilitate PD-L1 expression in dendritic cells as well as PD-1 and CTLA-4 expression in CD8^+^ T cells, called cytotoxic T lymphocytes (CTL), to become exhausted CD8^+^ T cells. TAM also promote angiogenesis, invasion, and intravasation, and, at the metastatic site, extravasation of tumor cells and persistent growth, and it also suppresses cytotoxic T cell responses [146]. In this context, TAMs and Treg are potential anti-tumor targets that impair activation, survival, and expansion of tumors. T cells function through the production of immunosuppressive cytokines, such as TGF-β, CTLA-4, and interleukin 10 [147].

CXCR4 is known to accelerate T cell proliferation and secretion of interferon γ, which facilitates PD-L1 expression in cancer cells and TAMs and also PD-1 expression in CTLs and natural killer cells [148,149]. Inhibition of CXCR4 suppresses Treg infiltration into the tumor and increases the anti-tumor immune response. Recent in vivo preclinical studies indicated that the combination of small molecule CXCR4 antagonists and anti-PD-1 antibodies significantly enhanced anti-tumor effects compared with single-agent administration in syngeneic murine models of ovarian cancer, colon cancer, and melanoma [150,151]. These effects were based on the prevention of immunosuppression in the tumor microenvironment characterized by the enhanced effector T cell and decreased Treg. The latter study with a human melanoma xenograft also indicated the possible inhibition of cancer cell-intrinsic PD-1 and T cell-independent responses, providing relevant information regarding combination therapy for enhancing anti-tumor immunity [151].

## 11. CXCR4-Directed Imaging and Anti-Cancer Therapy with α Particle Radiation

CXCR4 antagonist plerixafor has been approved as a stem cell mobilization agent for peripheral stem cell collection for the autologous peripheral stem cell transplantation [152], and ^68^Ga-pentixafor has been used for CXCR4-directed PET imaging in patients with hematologic neoplasms and malignant solid tumors [153,154]. CXCR4 is one of the most prevalent chemokine receptors ubiquitously expressed in cancer cells. Since CXCR4 is known to be expressed in various CSCs and is associated with tumorigenicity, angiogenesis, invasion, and chemoresistance [144], CXCR4-directed therapies have been exploited using either alternative small-molecule CXCR4 antagonists [155], CXCL12 inhibitors [156], or anti-CXCR4 antibodies [157]. Radiolabeled compounds targeting CXCR4 have also been examined with lutetium-177 labeled pentixather, copper-64 labeled plerixafor, and another small molecule [158,159,160]. Fluorine-18 labeled pentixafor analog has recently shown high CXCR4 affinity and favorable tumor-to-normal organ ratios in a murine model of human lymphoma [161]. Emerging approaches for CXCR4-targeted imaging as above have shown promise in addressing the need for monitoring future CXCR4-targeted therapy.

Functionalized gold nanoparticles containing reniumu-186 and coupled with anti-CXCR4 antibodies have been developed to actively target CSCs to reverse chemoresistance in an orthotopic mouse model of glioblastoma [162]. In addition to ordinary radionuclide therapy using β^−^ particles emission, as mentioned above, targeted α particle therapy (TAT) has great potential for the treatment of cancer on the basis of specific delivery of high-energy radiation from α-emitting radionuclides to tumors while minimizing systemic toxic effects. TAT has been regarded to have alternative treatment options for advanced or refractory cancers [163,164]. The initial results of clinical trials with ^213^Bi- and ^225^Ac-labeled compounds were attractive even in cases refractory to β-emitters [165,166]. The high linear energy transfer (~100 keV/µm) of α particles, in comparison with β^−^ particles, requires fewer radiation tracks to induce DNA double strand breaks. The short path-length of α particle within a few cell diameters confines its cytotoxic effect to the target tissue and the surrounding short range of the tumor microenvironment while limiting its toxic effects to non-neoplastic tissues. Moreover, the effect of oxygenation on cytotoxicity is minimal; thus, effective cell death can be expected even in the areas of hypoxia.

Among α particle emitting radionuclides for TAT, astatine-211 is promising because it can be produced using cyclotron by the bombardment of an α particle beam, and the procedures of separation and purification from the target has been established. Astatine-211 emits α particles by a 100% probability with a half-life of 7.21 h. There are a few clinical applications of astatine-211 which have been reported for the therapy of malignant neoplasms [167,168]. Since the escape of stem cells from anti-cancer therapy is considered a major cause of treatment failure and relapse, CXCR4-directed therapy with preserved anti-tumor immunity in the tumor microenvironment would be potentially effective. For this reason, we have explored stem-cell-targeted radioimmunotherapy with α particles in AML [169].

## 12. Response Evaluation of Novel Therapeutics with Molecular Imaging

Malignant cells survive in a complex balance in the immune system. Both CTLA-4 and PD-1 suppress T cell activities. Therefore, agents that block CTLA-4, PD-1, and PD-L1 are able to produce an anti-tumor response through immune activation. Inhibition of CXCR4 exaggerates the anti-tumor immune response and CXCR4-targeted therapy is a possible therapeutic option to eradicate CSCs. Recent studies have indicated that dual blockade of PD-1–PD-L1 and CXCL-12–CXCR4 pathways reduces specific cellular and functional elements within the immunosuppressive tumor microenvironment and augments tumor-specific cell-mediated immune responses. The complexity of these interactions and heterogeneity of immune cells in the tumor microenvironment are challenges in the development and the evaluation of the therapeutic efficacy of new immune therapies in vivo. Imaging of immune cells that are major players in anti-cancer therapy is challenging because many subtypes of cells exist and play different roles in the tumor microenvironment.

Non-invasive evaluation procedures for therapy outcomes, such as biomarkers and molecular imaging, are expected to represent precise strategies of cancer therapy. FDG-PET can play an important role in fulfilling this purpose, as mentioned earlier [38,42,100]. Uptake of FDG reflects the viability of cancer cells and all other players of the immune system in the microenvironment. No uptake of FDG means complete remission of the tumor; however, increased uptake does not always indicate progression of the tumor, because of the pseudoprogression phenomenon and increased anaerobic glycolysis in the therapy-induced hypoxia, as mentioned above [18,38,114]. Cancer cell-specific imaging has the potential to evaluate quantitatively the residual cancer cells that had been able to evade anti-cancer agent of immune response. However, phenotypic changes due to genetic alteration, such as therapy resistant mutation and de novo mutation after therapy, may decrease specificity to the specific imaging agent. Metabolism-based PET tracers other than FDG can be used to evaluate therapeutic efficacy [98,99]. However, metabolic diversity and instability, especially those acquired on the progression course or after therapy, of cancer cells would be sources of inaccuracy in evaluating the response.

Prediction and evaluation of therapeutic efficacy would be possible with a tumor-specific PET tracer. Prostate-specific membrane antigen (PSMA) ligand labeled with gallium-68 (^68^Ga-PSMA) is a PET tracer used to determine the eligibility for PSMA-targeted radionuclide therapy with ^177^Lu-PSMA or ^235^Ac-PSMA (Table 2). Peptide receptor radionuclide therapy for neuroendocrine carcinoma with ^90^Y- and ^177^Lu-dodecane-tetraacetic acid-Tyr^3^-octreotate (DOTA-TATE) is another radionuclide therapy performed successfully for solid tumors. ^68^Ga-DOTA-TATE is a diagnostic counterpart of therapeutics. These examples are representative theranostics in nuclear medicine practice that will be followed by the future radionuclide therapy. A major role of specific imaging in the theranostics is to confirm the indication of therapy. Another role would be dosimetry analysis to determine the therapeutic dose by calculating absorbed doses in the tumor for efficacy and target organs for toxicity. It may be possible for PET imaging with specific tracers to evaluate therapeutic efficacy by measuring the amount of target molecules; however, the expression of the target molecules may change after the therapy—then, accurate response evaluation would be difficult with these target-specific PET studies.

Considering the present availability and required standardization, FDG-PET may be favorable for response evaluation in solid tumors. Since there is a variety of therapeutics that have effects on both cancer cells and the immune system, individualized evaluation criteria based on therapeutic agents and clinicopathologic information may be appropriate. Clinicopathologic data include therapeutic regimen and time from administration, immune function status, temporal changes in size and attenuation of tumor on CT, and pathological parameters, such as proliferation, invasion, differentiation, vascularity, and interstitial findings. These data as well as image features and quantitative indices like SUV and MTV of PET are subjected to artificial intelligence (AI) for radiomics analysis. Other available data such as MRI and contrast enhancement are welcome by AI for more detailed analyses.

Modalities used in the clinical setting include PET and single photon emission computed tomography, as well as MRI and ultrasonography. Optical imaging, such as fluorescence and bioluminescence imaging, plays an important role in preclinical settings; however, penetration of these signals is too shallow to detect labeled immune cells in clinical situations, and currently used contrast materials, such as gadolinium based agents, super paramagnetic iron oxide, and perfluorocarbon labeled with fluorine-19, for MRI are non-specific for immune cells. Therefore, nuclear medicine imaging is a possible procedure to elucidate anti-cancer immune responses [170,171]. Cell tracking of particular cell subsets would be done by radiolabeling in vitro prior to re-administration or by injecting a radiopharmaceutical that binds to a specific membrane antigen in vivo [172,173]. There have been many radiopharmaceuticals for cell tracking; however, none of these have been successfully used in clinical practice so far (Table 3).

## 13. Conclusions

Quantitative parameters of FDG-PET, such as SUV and TLG, have been used to evaluate therapeutic responses. Metabolic changes and temporal enlargement due to immune cell infiltration seen after immune checkpoint inhibitors, anti-PD-1, and anti-PD-L1 antibodies facilitate the modification of FDG-PET response evaluation criteria as well as conventional RECIST. Dynamic interaction between cancer and immune cells, CSCs, and metabolism of cancer cells in the tumor microenvironment are promising targets to eradicate cancer. Accumulation of FDG reflects glucose metabolism of both cancer cells and immunologically competent cells in the tumor. Considering inter- and intra-patient tumor heterogeneity, immunological reaction to the therapy differs among patients according to the individual immune function and tumor heterogeneity. This limits the use of current response evaluation criteria and the revised ones may not be relevant enough for use in the clinical setting. Then, imaging of immune cells tracking may be crucial but is still a challenge, due to the fact that radiopharmaceuticals or MRI probes which are highly specific for biomarkers expressed in different immune cells are not likely to be determined. A radiomics approach which combines quantitative image features and deep learning algorithms has the potential to improve response assessment on the basis of elucidating pathologic mechanisms in more personalized treatments in the era of precision nuclear medicine. Multimodal imaging to highlight new therapeutic biomarkers in the complexed tumor response may be required to improve the management of cancer patients.

## Figures and Tables

**Table 1:** Conventional/Immune-Related Tumor Response Evaluation Criteria and 2-[^18^F] fluoro-2-deoxy-d-glucose positron emission tomography (FDG-PET) Response Evaluation Criteria.

Conventional and Immune-Related Tumor Response Criteria						
Criteria	Measurement	CR	PR	PD	Confirmation of PD	New Lesion
RECIST 1.1 (2009)	Unidimensional (LD for non-nodal lesions; LPD for LN)	Disappearance of all target lesions <10 mm for any pathological LN	≥30% reduction	≥20% and ≥5 mm increase, new lesion, or nontarget PD	Not required	Defines PD
irRECIST (2013)	Unidimensional (LD for non-nodal lesions; LPD for LN)	Disappearance of all target lesions	≥30% reduction	≥20% and ≥5 mm increase, new lesion, or nontarget PD	Required on consecutive studies at least 4 weeks apart	Does not define PD; measurements of new lesions included in the total tumor burden
iRECIST (2017)	Unidimensional (LD for non-nodal lesions; LPD for LN)	Disappearance of all target lesions	≥30% reduction	≥20% and ≥5 mm increase, new lesion, or nontarget PD	Required at the next assessment 4–8 weeks later	Defines unconfirmed PD; confirms PD if additional new lesions or size increase (≧5 mm for the sum of new target or any increase in new nontarget lesions) are noted on the next assessment
**FDG-PET Response Evaluation Criteria**						
**Criteria**	**Measurement**	**CMR**	**PMR**	**PMD**	**Confirmation of PMD**	**New Lesion**
EORTC (2000)	SUVmax	Complete resolution of FDG uptake in all lesions	> 25% reduction in the sum of SUVmax after more than one cycle of treatment	> 25% increase in the sum of SUVmax or appearance of new lesions	Not required	Defines PD
PERCIST (2009)	SUVpeak	Complete resolution of FDG uptake in all lesions	≥ 30% reduction of the peak lean body mass SUV (SULpeak) and an absolute drop of 0.8 SULpeak units	> 30% increase in the SULpeak and an absolute increase of 0.8 SULpeak, or appearance of new lesions	Not required	Defines PD

RECIST: response evaluation criteria in solid tumors; irRECIST: immune-related RECIST; iRECIST: immune RECIST; EORTC: the European Organization for Research and Treatment of Cancer; PERCIST: PET Response Criteria in Solid Tumors; LD: longest diameter; LPD: longest perpendicular diameter; LN: lymph nodes; SUVmax: maximum standardized uptake value; SUVpeak: peak standardized uptake value; SULpeak: peak lean body mass standardized uptake value; CR: complete response; PR: partial response; PD: progressive disease; CMR: complete metabolic response; PMR: partial metabolic response; PMD: progressive metabolic disease.

**Table 2:** Representative Pair of Radiopharmaceuticals for Theranostics.

Radiopharmaceutical for Therapy	Radiation	Half-Life	Radiopharmaceutical for Diagnosis
^177^Lu-DOTA-TATE	Beta ray (β^−^ particle)	78 h	^68^Ga-DOTA-TATE
^213^Bi-DOTA-TOC	Alpha ray (He^2+^ particle)	0.76 h	^68^Ga-DOTA-TOC
^177^Lu-PSMA	Beta ray (β^−^ particle)	78 h	^68^Ga-PSMA
^225^Ac-PSMA	Alpha ray (He^2+^ particle)	10 d	^68^Ga-PSMA

DOTA-TATE: dodecane-tetraacetic acid-Tyr^3^-octreotate; DOTA-TOC: dodecane-tetraacetic acid-D-Phe^1^-Tyr^3^-octreotide; PSMA: prostate-specific membrane antigen.

**Table 3:** Potential Radiopharmaceuticals to Image Immune Cells and Cell Tracking.

Target	Radiolabeling Agent	Application/Mechanism	References
T lymphocytes	^111^In-oxine, ^89^Zr-oxine	Tumor infiltration	[171,174,175]
	^18^F-FDG	Cytokine production	
	SPIO		
NK cells	^111^In-oxine, ^89^Zr-oxine	Tumor infiltration	[171,176,177]
	^18^F-FDG, ^11^C-methyl iodide	NK cell homing	
	SPIO		
Macrophages	^111^In-oxine, ^89^Zr-nanoparticles	Tumor infiltration	[171,178,179,180,181,182]
	^18^F-FDG	Tumor-associated macrophages	
	SPIO, ^19^F-perfluorocarbon		
Interleukin-2	Iodine-123, Technetium-99 m, Fluorine-18	Interleukin-2 receptors on T cells	[183]
Anti-CD8 cys-diabody	Zirconium-89, Copper-64	CD8^+^ T cells	[184,185,186]
Anti-CD8 mAb			
PK11195	Carbon-11	Tumor-associated macrophages, Translocator protein	[187]
Anti-TCR mAb	Copper-64	Tumor infiltration of T cells	[188]
Anti-CD56 mAb	Technetium-99 m	NK cells	[189]

^18^F-FDG: 2-[^18^F] fluoro-2-deoxy-d-glucose; SPIO: super paramagnetic iron oxide; TCR: T cell receptor; mAb: monoclonal antibody.
